# Chronic Lead Exposure Increases Blood Pressure and Myocardial Contractility in Rats

**DOI:** 10.1371/journal.pone.0096900

**Published:** 2014-05-19

**Authors:** Mirian Fioresi, Maylla Ronacher Simões, Lorena Barros Furieri, Gilson Brás Broseghini-Filho, Marcos Vinícius A. Vescovi, Ivanita Stefanon, Dalton Valentim Vassallo

**Affiliations:** 1 Department of Physiological Sciences, Federal University of Espírito Santo, Vitoria, Espírito Santo, Brazil; 2 Department of Nursing, Federal University of Espírito Santo, Vitoria, Espírito Santo, Brazil; 3 Health Science Centre of Vitória-EMESCAM, Vitória, Espírito Santo, Brazil; 4 Department of Chemistry, Federal University of Espírito Santo, Vitoria, Espírito Santo, Brazil; Temple University, United States of America

## Abstract

We investigated the cardiovascular effects of lead exposure, emphasising its direct action on myocardial contractility. Male Wistar rats were sorted randomly into two groups: control (Ct) and treatment with 100 ppm of lead (Pb) in the drinking water. Blood pressure (BP) was measured weekly. At the end of the treatment period, the animals were anaesthetised and haemodynamic parameters and contractility of the left ventricular papillary muscles were recorded. Blood and tissue samples were properly stored for further biochemical investigations. Statistical analyses were considered to be significant at p<0.05. The lead concentrations in the blood reached approximately 13 µg/dL, while the bone was the site of the highest deposition of this metal. BP in the Pb-treated group was higher from the first week of lead exposure and remained at the same level over the next four weeks. Haemodynamic evaluations revealed increases in systolic (Ct: 96±3.79 *vs*. Pb: 116±1.37 mmHg) and diastolic blood pressure (Ct: 60±2.93 *vs*. Pb: 70±3.38 mmHg), left ventricular systolic pressure (Ct: 104±5.85 *vs*. Pb: 120±2.51 mmHg) and heart rate (Ct: 307±10 *vs.* Pb: 348±16 bpm). Lead treatment did not alter the force and time derivatives of the force of left ventricular papillary muscles that were contracting isometrically. However, our results are suggestive of changes in the kinetics of calcium (Ca^++^) in cardiomyocytes increased transarcolemmal Ca^++^ influx, low Ca^++^ uptake by the sarcoplasmic reticulum and high extrusion by the sarcolemma. Altogether, these results show that despite the increased Ca^++^ influx that was induced by lead exposure, the myocytes had regulatory mechanisms that prevented increases in force, as evidenced in vivo by the increased systolic ventricular pressure.

## Introduction

According to the World Health Organization, cardiovascular diseases are currently the leading cause of morbidity and mortality worldwide [1], and exposure to toxic substances, including lead and other metals, may contribute to the appearance or aggravation of these pathological processes.

Lead is a metal that is used to manufacture products that are used by humans. Currently, lead is used in the manufacturing of high-tech products for the protection of nuclear reactors, thin sheets of electronic components, batteries, paints, ceramics, cables and ammunition. Due to its extensive industrial use, lead continues to be released into the environment, and given that lead cannot be degraded, it contaminates the earth’s crust and drink water, thereby exposing the population to its toxic effects, remaining potentially noteworthy in the context of epidemiological impact [2], [3].

Several clinical and epidemiological studies suggest that there is an association between lead exposure and high blood pressure, and the evidence is sufficient to infer a causal relationship between these two variables [4], [5].

Using the database of the second *National Health and Nutrition Examination Survey* (NHANES II), which was conducted between 1976 and 1980, Pirkle et al. (1985) investigated the association between lead exposure and high blood pressure. They observed a positive association between blood lead concentrations and systolic and diastolic blood pressures [6]. However, not all studies have shown consensus when evaluating this association. For example, Vupputuri et al. (2003) studied 10 548 white men and 4404 black men and suggested that an association between blood lead concentrations and increased systolic and diastolic blood pressure existed in black, but not in white, men [7].

One hundred years of studies have correlated lead exposure with cardiovascular system function. However, the contribution of this metal has not yet been completely elucidated [5], which is largely because many factors may serve as isolated risk factors for cardiovascular diseases.

Several studies using animal models of chronic exposure have shown that lead exposure is a risk factor for the development of hypertension [8], [9], [10], [11], [12]. However, exposure to low concentrations of lead, for short periods of time was also recently shown to increase arterial pressure in animals [13], [14].

In addition, only a few studies have investigated the cardiac effects of lead exposure. Isolated right ventricle strips that were subjected to acute exposure to 20 µM lead showed reductions in isometric force and myosin ATPase activity [15].

Due to the lack of studies investigating the direct effects of low lead concentrations on the cardiac contractile machinery and the lack of knowledge regarding the mechanisms underlying the effects of lead on blood pressure, the direct interference of lead on myocardial contractility and the mechanisms by which it causes these changes were investigated. The present study aimed to evaluate cardiovascular impairment resulting from exposure to lead for 30 days and to investigate the possible mechanisms that render lead harmful to myocardial contractility, contributing to the deleterious effects of this metal on the cardiovascular system.

## Materials and Methods

### Animals

Studies were performed using male Wistar rats. All experiments were conducted in compliance with the guidelines for biomedical research as stated by the Brazilian Societies of Experimental Biology and were approved by the Institutional Ethics Committee of the Health Science Centre of Vitória (CEUA-EMESCAM 007/2007 and 003/2007). The rats were housed at a constant room temperature, humidity, and light cycle (12∶12 hr light-dark) with free access to water and were fed rat chow *ad libitum*.

When the rats reached two months of age, they were divided into two groups. One group of animals, known as the lead-treated group, was administered 100 ppm lead acetate (Sigma Chemical Co., USA) in their drinking water for 30 days (Pb-group). The other group of rats, which served as controls, was treated in the same manner as the lead-treated group, except that they were administered tap water (Ct-group).

At the end of the treatment period, the rats were anesthetised with urethane (1.2 mg/kg *i.p*.) for haemodynamic measurements. After this procedure, the animals were killed in order to investigate myocardial contractility, and the hearts were rapidly removed in order to permit dissection of the left ventricular papillary muscles. For the protein expression and Na^+^, K^+^–ATPase (NKA) activity analyses, the hearts were rapidly frozen in liquid nitrogen and stored at −80°C until analysed. Blood samples were collected in tubes with EDTA, placed on ice and centrifuged at 3 500×*g* for 15 min at 4°C. The plasma was stored at −80°C for later determinations of angiotensin converting enzyme (ACE) activity. Sample tissues (lung, kidney, heart, aorta, bone and brain) were frozen at −20°C and the blood samples were stored at –4°C for later analyses of lead concentrations in these tissues.

### Blood and Tissue Lead Level Measurements

Blood and tissue lead level measurements were determined according to the protocol developed by Korecková-Sysalová (1997) [16]. Lead concentrations in the whole blood and tissue samples after 30 days of treatment were measured in duplicate using atomic spectrometry (model: AAS5 EA with graphite furnace, Carl Zeiss, Germany).

### Blood Pressure Measurements with Conscious Rats

Indirect systolic blood pressure was measured weekly, and at the beginning and end of treatment, using tail-cuff plethysmography (IITC Life Science, Inc, Woodland Hills, CA, USA). Conscious rats were restrained for 5–10 min in a warm, quiet room and conditioned to numerous cuff inflation-deflation cycles by a trained operator. Systolic blood pressure was reported as the arithmetic mean of three recorded measurements.

### Haemodynamic Measurements

At the end of the treatment period, the rats were anaesthetised with urethane (1.2 mg/kg i.p.), and the carotid artery and jugular vein were cannulated with a polyethylene catheter (PE-50/*Clay-Adams*) and filled with heparin (50 U/ml) in saline. The cannulae were connected to pressure transducers (TSD 104A- Biopac) connected to a preamplifier and acquisition system (MP 30 Biopac Systems, Inc; CA) for pressure measurements. The carotid artery and jugular vein cannulae were advanced into the left and right ventricular chambers for pressure measurements and recordings. The following parameters were analysed: systolic (SAP), diastolic (DAP) and mean (MAP) arterial pressures; left and right ventricular systolic pressures (LVSP and RVSP); left and right ventricular end-diastolic pressures (LVEDP and RVEDP); positive (+) and negative (−) time derivatives of the left and right ventricular pressures (dP/dt) and heart rate (HR).

### Isometric Contractility Studies Using Papillary Muscles

After the haemodynamic experiments had been completed, the anaesthetised animals were killed, the thoraxes were opened and the hearts were rapidly removed. The hearts were perfused through the aortic stumps with modified *Krebs* solution (which had the following composition (in mM): 120 NaCl; 5.4 KCl; 1.25 CaCl_2_; 1.2 MgCl_2_; 2 NaH_2_PO_4_; 1.2 Na_2_SO_4_; 24 NaHCO_3_; 11 glycose; pH = 7.4) in order to permit the proper selection and dissection of the left ventricular papillary muscles. The preparations were bathed in 20 ml of the gassed (95% O_2_ and 5% CO_2_) solution and maintained at 26±0.5°C in order to avoid hypoxia [17]. The preparations were attached to an isometric transducer (TSD125– Biopac Systems, Inc; CA). Field stimulation was provided by isolated rectangular pulses (10 to 15 V, 12 ms duration) that were applied through a pair of platinum electrodes that were placed along the entire extension of the muscle. The standard rate of stimulation was 0.5 Hz. Records were started after 45 to 60 min in order to allow the beating preparation to adapt to the new environmental conditions. The force developed during contractions was measured in mg/mg (developed force, in mg, divided by muscle weight, in mg). A correction for the papillary weight was used to normalise the data from different preparations. The following protocols were used:

1. The effects of lead treatment on isometric force development were compared to those of control rats under control conditions.
2. Under steady-state conditions, the force was measured at different Ca^++^ concentrations (0.5, 1.0, 1.5 and 2.0 mM) in preparations from control and lead-treated rats.
3. Post-rest potentiation (PRP) was used to provide information about the function of the sarcoplasmic reticulum (SR). In cardiac muscle, the contractions that occurred after short pauses were potentiated, and these post-rest contractions were dependent upon pause duration and the amount of calcium that was stored at intracellular sites. Pause intervals of various durations (15, 30 and 60 s) were used, and the results are presented as the relative potentiation (the amplitude of the post-rest contractions divided by the steady-state contractions) in order to normalise the data from the different preparations [18].
4. The tetanic tension consists of a protocol to investigate the contractile response in the intact myocardium with a non-functional SR (19). The tetanic tension was elicited by high frequency stimulation (10 Hz for 15 s) and was achieved after 5 mM caffeine pretreatment for 30 min. The tetanic tension was assessed both before and after verapamil (10 µM) treatment. Verapamil was added to the bath 20 min before the next administration of tetanus [19].
5. Post rest contractions (PRC) were obtained after 10 min in the absence of stimulation. The muscles were maintained in a solution containing caffeine (5 mM), but not calcium (calcium free solution). To achieve the PRC, the calcium free solution was exchanged with by a modified Krebs solution (containing calcium, 1.25 mM) seconds before the beginning of electrical stimulation. The first contraction after rest was taken as an index of sarcolemmal calcium influx [20].

### Biochemical Analyses

#### Determination of angiotensin-converting enzyme (ACE) activity

The effects of lead treatment on the activity of serum angiotensin-converting enzyme (ACE) were determined using the procedure outlined by Oliveira et. al. (2000) [21]. Briefly, the serum (3 ml) was incubated with 40 ml of assay buffer containing 5 mM Hip-His-Leu in 0.4 M sodium borate buffer with 0.9 M NaCl, pH = 8.3, for 15 min at 37°C. The reaction was terminated by the addition of 190 ml of 0.34 N NaOH. The product, His-Leu, was measured fluorometrically at an excitation wavelength of 365 nm and an emission wavelength of 495 nm using a fluoro-colorimeter (Synergy 2, Biotek). Seventeen microlitres of o-phathaldialdehyde (20 mg/ml) in methanol were added. To correct for the intrinsic fluorescence of the serum, time zero blanks (T_o_) were prepared by adding serum after NaOH. All assays were performed in triplicate.

#### Determination of Na^+^, K^+^–ATPase (NKA) activity

The enzymatic material that was used to determine whether lead exposure is capable of affecting Na^+^, K^+^-ATPase activity was extracted as described by Stefanon et al. (2009) [22]. Ventricular tissue was homogenised in a solution containing Tris-HCl (20 mM) and EDTA (1 mM) at a pH of 7.5. The homogenised tissue was centrifuged at 10,000×*g* for 20 minutes and the precipitate was discarded. The same volume of Tris-HCl (20 mM) and EDTA (1 mM) at a pH of 7.5 was added to the supernatant and centrifuged at 10,500×*g* for 1 h. The precipitate was resuspended in Tris-HCl (20 mM) and EDTA (1 mM) at a pH of 7.5 in a final volume of 400 µL.

Na^+^, K^+^-ATPase activity was assayed by measuring Pi liberation from 3 mM ATP in the presence of NaCl (125 mM), MgCl_2_ (3 mM), KCl (20 mM) and Tris-HCl (50 mM) (pH = 7.5). The enzyme was pre-incubated for five minutes at 37°C, and the reaction was initiated by adding ATP. Incubation time and protein concentration were chosen to ensure the linearity of the reaction. The reaction was stopped by the addition of 200 µL of 10% trichloroacetic acid. Controls, with the addition of the enzyme preparation after the addition of trichloroacetic acid, were used in order to correct for non-enzymatic hydrolysis of the substrate. All samples were performed in duplicate. The specific activity was reported as nmol of Pi released per min per mg of protein, unless otherwise stated. The specific activity of the enzyme was determined in the presence and absence of 5 mM ouabain.

### Western Blot Analyses

#### Western blot analyses

After the experiments, the hearts were homogenised and the proteins (80 µg) were separated using 10% SDS-PAGE gels for the quantification of the SR Ca^++^-ATPase (SERCA-2a), sodium-calcium exchange (NCX) and the α-1 Na^+^, K^+^-ATPase subunit. The low molecular weight proteins phospholamban (PLB), phospho-Ser^16^-PLB and phospho-Tre^17^-PLB were separated using 15% SDS-PAGE gels.

The proteins were transferred to nitrocellulose membranes, which were incubated with mouse monoclonal antibodies for SERCA 2a (1∶1000, Thermo Scientific, Rockford, USA), NCX (1∶1000, Thermo Scientific, Rockford, USA), Na^+^, K^+^-ATPase α-1 (1∶500, Millipore, San Francisco, U.S.A.), PLB (1∶1000, Thermo Scientific, Rockford, USA), phospho-Ser^16^-PLB (1∶5000, Badrilla, West Yorkshire, UK) or phospho-Tre^17^-PLB (1∶5000, Badrilla, West Yorkshire, UK). After being washed, the membranes were incubated with an anti-mouse (1∶5000, Sigma Chemical, Co., St Louis, U.S.A.) immunoglobulin antibody that was conjugated to horseradish peroxidase. After being thoroughly washed, the immunocomplexes were detected using an enhanced horseradish peroxidase/luminal chemiluminescence system (ECL Plus, Amersham International, Little Chalfont, UK) and film (Hyperfilm ECL International). The signals on the immunoblot were quantified using the National Institutes of Health Image V1.56 computer program. The same membrane was used to determine GAPDH (Glyceraldehyde 3-phosphate dehydrogenase) expression using a rat monoclonal antibody for GAPDH (1∶5000, Abcam Cambridge MA, USA.). After being washed, it was incubated with an anti-mouse antibody (1∶5000, Assay Designers, Hines Drive, Ann Arbor, MI). All reagents for western blotting were purchased from Sigma Chemical Co., USA.

### Drugs Used

The following drugs were used: heparin (Roche Q.F.S.A., Brazil), anhydrous caffeine (B. Herzog, Brazil), urethane, bovine serum albumin, lead acetate and verapamil (Sigma Chemical Co., USA). All of the other reagents used were of analytical grade and were obtained from Sigma, E. Merck (Germany) or Reagen (Brazil).

### Data Analyses and Statistics

All values are expressed as the means ± SEM of the number of animals used in each experiment. The results were analysed using the completely randomised Student’s t-test, or one-way ANOVA or two-way ANOVA. When ANOVA showed a significant treatment effect, Tukey’s or Bonferroni’s post tests was used to compare the individual means. Differences were considered to be statistically significant at p<0.05. The data were analysed and the figures were plotted using GraphPad Prism (Version 2.0, GraphPad Software, USA).

## Results

### Body Weights and Blood and Tissue Lead Concentrations

Pb exposure did not affect the body weights of the animals. At the end of the treatment period, the body weights were similar between the groups (Ct = 335±11 g, Pb = 336±12, n = 10). Moreover, the Pb levels in the blood were increased in the Pb-group (Ct <0.5 µg/dL, Pb = 13.6±1.07 µg/dL, n = 6). The metal accumulated to higher levels in the tissues of the Pb-group compared to the Ct-group, with the exception of the brains, which showed similar concentrations in both groups (Table 1). Evaluation of lead concentrations in the pancreas, spleen and testes indicated that these organs are sites of low metal deposition, since the lead concentration, in treated animals, did not exceed 0,04 µg/g in any of these organs.

**Table 1:** Tissue lead concentrations after 30 days of lead treatment (100 ppm).

Pb (µg/g)	Ct-group	Pb-group	n
**Kidneys**	0.22±0.04	3.73±0.36*	7
**Heart**	0.21±0.07	0.57±0.09*	7
**Bone**	0.23±0.07	12.69±2.38*	7
**Lungs**	0.12±0.02	0.32±0.04*	7
**Brain**	0.21±0.09	0.18±0.03	7
**Aorta**	0.02±0.00	0.06±0.01*	7

Means ± SEM. Number of animals = 7 each group.

*p<0.05, as assessed using Student’s t*-*test.

The lead distribution proportional to each tissue mass was assessed after 30 days of lead exposure. Among the organs evaluated, bone tissue showed a value corresponding to 73% of the lead that was deposited. The second highest deposition site was the kidneys, with 23% of the lead that was deposited. The heart and lungs, with 3% and 2% of the lead that was deposited, ranked third and fourth, respectively, among the target organs following lead exposure. The brain and vascular tissues contained only 1% of the lead that was deposited.

It is important to mention that despite the exposure of the animals to lead, chronic intoxication with levels that were below the reference limits did not affect the weights of the animals.

### Blood Pressure and Ventricular Parameters

**Figure 1:**
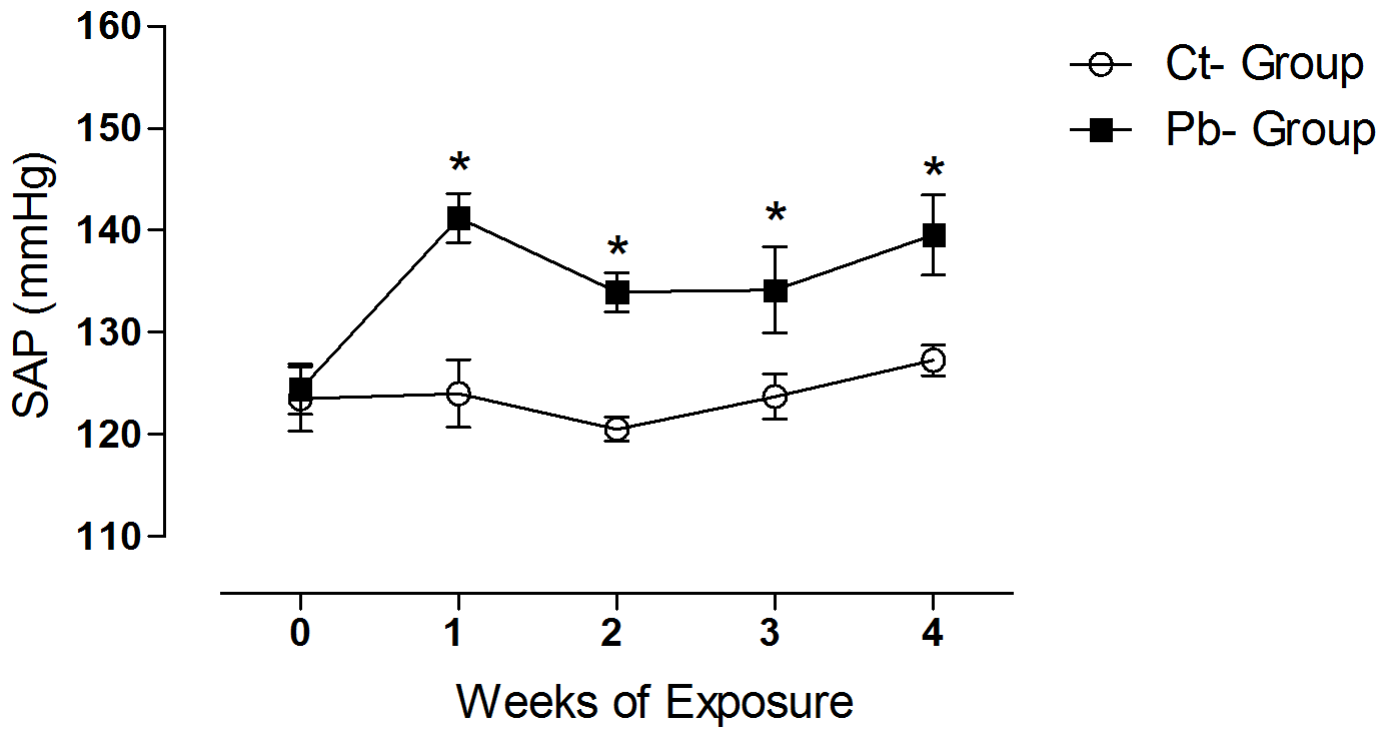
Systolic Arterial Pressure, obtained by tail plethysmography before (0) and during a 4 week period of Pb exposure. Symbols represent mean ± SEM (N = 7 for each group). Open circles- controls, filled squares- lead treated group. *p<0.05, Ct-group *vs.* Pb-group, two-way ANOVA, followed by Bonferroni’s test.

The systolic blood pressure was similar in both groups at the beginning of the treatment period. However, from the first week of metal exposure onward, the treated group exhibited an increase in arterial pressure that was not observed in the control group. Systolic blood pressure in the animals that had been treated with lead remained high over the following weeks. At the end of the treatment period, the SAP was approximately 127 and 140 mmHg, respectively, for the control and treated groups (Figure 1).

The arterial and ventricular parameters were assessed *in vivo* using haemodynamic measurements of anaesthetised animals at the end of 30 days of Pb exposure. The Pb-group exhibited an increase in SAP, DAP, MAP and HR (Table 2). The assessment of left ventricular (LV) performance (Table 1) revealed that Pb exposure increased LVSP, as well as the left ventricular dP/dt positive and negative, which are indices of contractility and relaxation, respectively.

**Table 2:** Effects of lead exposure on haemodynamic arterial and ventricular parameters in anaesthetised rats.

	Ct-group	n	Pb-group	n
**SAP(mm Hg)**	96±3.79	12	116±1.37*	11
**DAP(mm Hg)**	60±2.93	12	70±3.38*	10
**MAP(mm Hg)**	74±3.34	12	86±2.19*	10
**LVSP(mm Hg)**	104±5.85	10	120±2.51*	12
**LVDP(mm Hg)**	2.56±0,67	10	2.33±0,71	12
**dP/dt+LV(mm Hg/s)**	3795±332	10	5558±235*	12
**dP/dt – LV(mm Hg/s)**	−4928±253	9	−6464±295*	12
**RVSP(mm Hg)**	30±1.56	9	30±1.73	9
**RVDP(mm Hg)**	1,85±0.67	9	0,94±0.50	9
**dP/dt+RV(mm Hg/s)**	1363±200	9	1480±209	9
**dP/dt – RV(mm Hg/s)**	−1550±134	9	−1689±147	9
**HR(bpm)**	307±10	12	348±16*	12

Systolic Arterial Pressure (SAP); Diastolic Arterial Pressure (DAP); Mean Arterial Pressure (MAP); Left Ventricular Systolic Pressure (LVSP); End Left Ventricular Diastolic Pressure (LVDP); Right Ventricular Systolic Pressure (RVSP); End Right Ventricular Diastolic Pressure (RVDP); Left Ventricular Positive (dP/dt+LV) and Negative (dP/dt – LV) Derivates; Right Ventricular Positive (dP/dt+RV) and Negative (dP/dt – RV) Derivates; and Heart Rate (HR). Means ± SEM. Animal number range = 9–12.

*p<0.05, as assessed using Unpaired Student’s t-test.

Unlike the results observed in the LV, treatment with lead did not modify right ventricular parameters (Table 2).

### Myocardial Contractility

At the end of the lead treatment period, although lead exposure had increased SAP and LVSP, the contractile force of isolated papillary muscles remained unchanged (Ct = 404.7±36,4 mg/mg, Pb = 422.2±44.9 mg/mg, n = 10 and 12, respectively, p = 0.772). The force derivatives, dF/dt positive (Ct = 2305±225 mg/mg/s, Pb = 2364±225 mg/mg/s, n = 10 and 12, respectively, p = 0.856) and negative (Ct = −4271±660 mg/mg/s, Pb = −4254±531 mg/mg/s, n = 10 and 12, respectively, p = 0.984) also remained unchanged after 30 days of Pb exposure. When evaluating the contractile effects of Pb on temporal parameters, both time to peak (Ct = 204±9.5 ms, Pb = 198±4.7 ms, n = 10 and 12, respectively, p = 0.557) and relaxation time (Ct = 557±30.4 ms, Pb = 556±39.2 ms, n = 10 and 12, respectively, p = 0.976) remained unchanged.

Other protocols were used to analyse the effects of lead treatment on the cellular mechanisms responsible for heart inotropism. These protocols investigated the concentration-response curves of increasing extracellular [Ca^++^], PRP, PRC and tetanic tension.

Pause intervals of various durations (15, 30 and 60 s) were used to provide indirect information about the function of the SR. Results are presented as relative potentiation (PRP). There is no difference in PRP values between Pb-group and Ct-group: PRP-15 s (Ct: 2.14±0.17 *vs.* Pb: 2.52±0.29), PRP-30 s (Ct: 2.62±0.27 *vs.* Pb: 2.95±0.37) and PRP-60 s (Ct: 2.76±0.31 *vs.* Pb: 3.39±0.47). These results suggested that lead treatment did not cause changes in sarcoplasmic reticulum activity.

**Figure 2:**
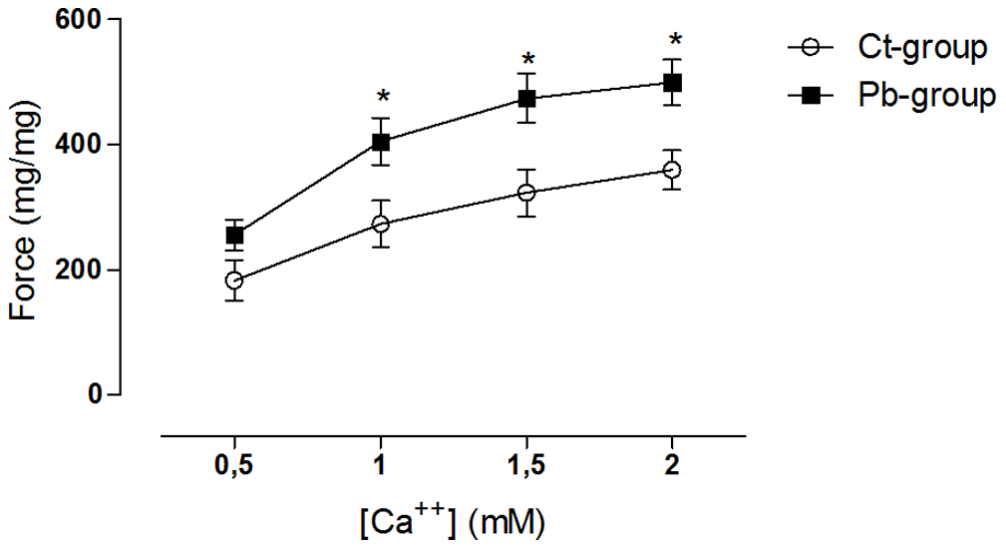
Concentration-response curve for increasing extracellular [Ca^++^] in isolated left ventricular papillary muscles from control and lead-treated rats. Symbols represent mean ± SEM. Open circles- controls (N = 9), filled squares- lead treated group (N = 10). *p<0.05, Ct-group *vs.* Pb-group, two-way repeated measures ANOVA, followed by Bonferroni’s test.

Another assay that was performed with the intention of investigating the effects of lead on the regulatory mechanism governing myocardial contractility consisted of the development of a concentration-response curve to examine changes in extracellular calcium concentrations. Figure 2 shows the largest papillary contractile response of animals in the lead treated group compared to the control group. This response suggests that either the cardiomyocytes of animals in the treated group are more permeable to calcium or their contractile proteins have a higher affinity for this ion [19].

**Figure 3:**
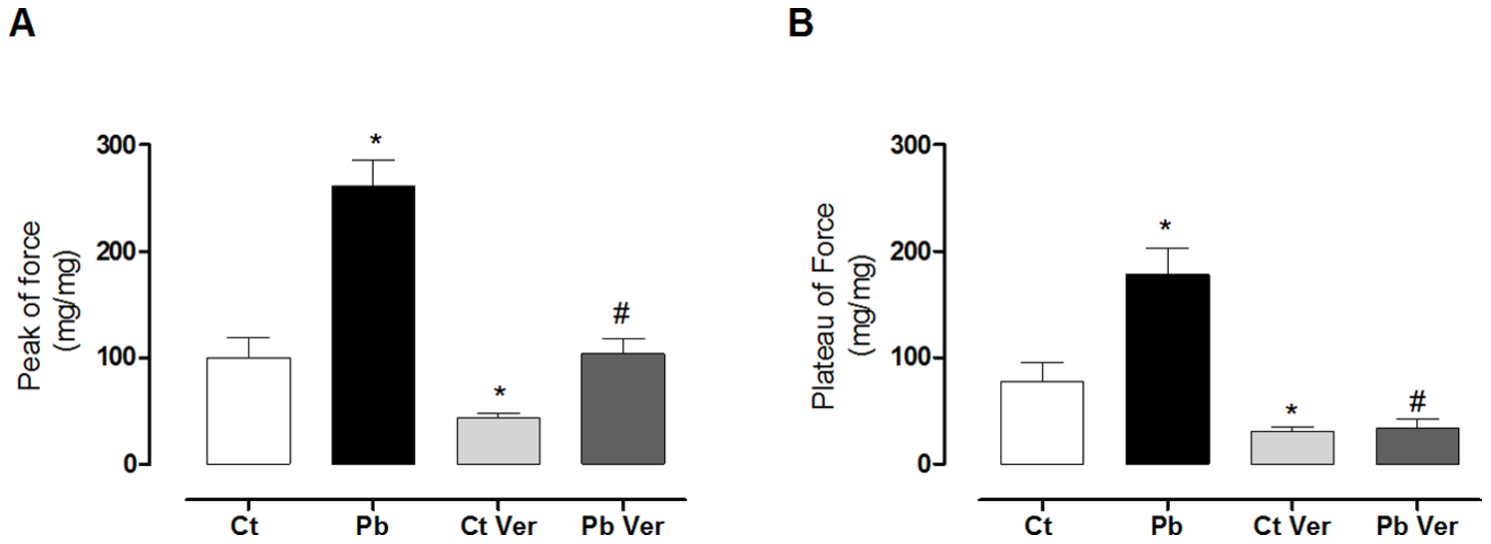
Peak and plateau of the isometric force in isolated left ventricular papillary after tetanic stimulation in the absence and presence of verapamil (Ver) of control (Ct), lead treated (Pb), control+verapamil (Ct Ver), lead treatred+verapamil (Pb Ver). Each column represents the means ± SEM. Number of animals = 6 for each group. *p<0.05, Pb *vs.* control, one-way ANOVA, followed by Tukey’s test.

The contractions obtained with PRC were performed to indirectly investigate calcium influx. The PRC was increased approximately 50% in lead exposure. Tetanic contractions are also dependent upon transarcolemmal calcium influx and on the sensitivity of contractile proteins to calcium because SR activity is blunted [19]. Figure 3 shows the increases in peak and plateau tetanic contractions that were promoted by lead exposure. These increases were abolished when the preparations were exposed to verapamil, a calcium channel blocker [23]. These findings corroborate the responses that were obtained after exposure to increasing calcium concentrations and PRC protocols, suggesting that the cardiomyocytes that were isolated from animals that were treated with lead have increased calcium influx when compared to the control group.

### Biochemical Measurements

#### Angiotensin-converting enzyme (ACE) activity

Previous reports have correlated the pressure effects of lead with activation of the renin-angiotensin system [12], [13], [14]. To investigate the involvement of this pathway in haemodynamic responses, ACE activity was measured in the plasma and cardiac tissue. Lead treatment did not alter the activity of this enzyme (Ct: 281.62±19.24; Pb: 275.7±18.33 nmol-His-Leu-min), suggesting that it was not involved in the changes that were promoted by lead exposure.

#### Na^+^, K^+^–ATPase (NKA) activity

NKA is an enzyme that regulates intracellular ion homeostasis by controlling the active transport of sodium and potassium in cells [24]. Lead treatment increased the activity of NKA (Ct: 138.2±43.45; Pb: 433.8±15.03 nmol Pi.min^−1^.mg protein^−1^) compared to the control group.

#### Western blot analyses

The quantification of important proteins that are involved in myocardial contractility regulation using Western Blot included analyses of the expression of SERCA-2, NCX, PLB, and phospho-Tre^17^-PLB and was corrected for total phospholamban (phospho-Tre^17^-PLB/PLB), phospho-Ser^16^-PLB, and phospholamban total (phospho-Ser^16^-PLB/PLB). The α-1 Na^+^, K^+^-ATPase subunit was used in order to investigate possible changes in calcium handling proteins.

**Figure 4:**
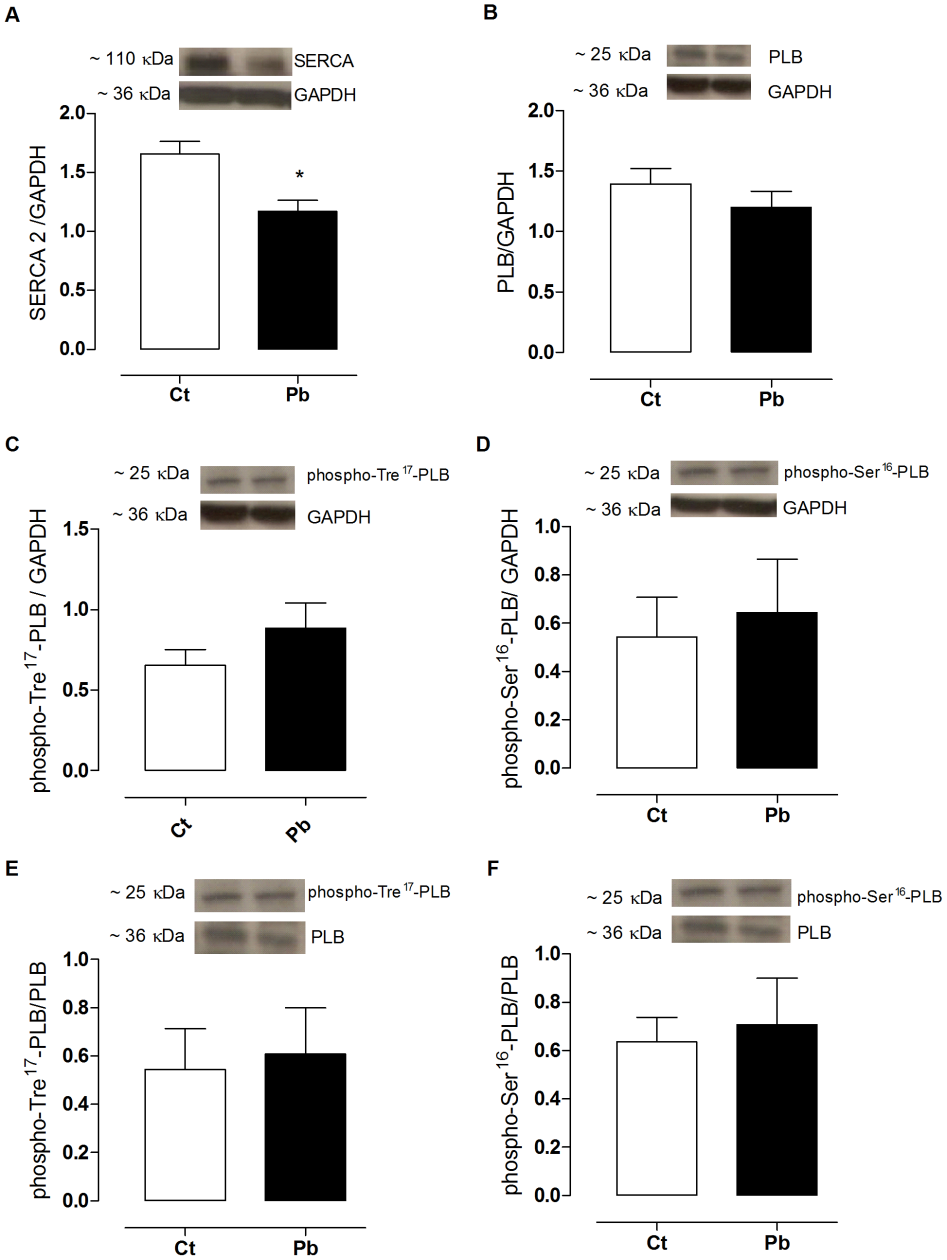
Protein expression levels of the ventricular calcium pump SR (SERCA 2), phospholamban (PLB), phospho-Tre^17^-PLB, phospho-Ser^16^-PLB, as well as the ratios phospho-Tre^17^-PLB/PLB and phospho-Ser^16^-PLB/PLB from controls (Ct) and lead treated (Pb) groups. Bands are representative of the Western blots of the expression of SERCA-2, PLB, phospho-Tre^17^-PLB, phospho-Ser^16^-PLB and GAPDH (Glyceraldehyde 3-phosphate dehydrogenase) are presented at the top of the figure. The results are expressed as the ratio between the area and density of SERCA-2 or PLB and GAPDH in the Ct- and Pb-groups. Number of animals = 7 for each group.*p<0.05, Ct vs Pb, Student t*-*test.

Figure 4 showed that treatment with lead reduced the expression of ventricular SERCA-2. However, treatment with lead did not alter the expression of PLB. The phospho-Tre^17^-PLB and phospho-Ser^16^-PLB, as well as the ratios of PLB total, remained unchanged.

**Figure 5:**
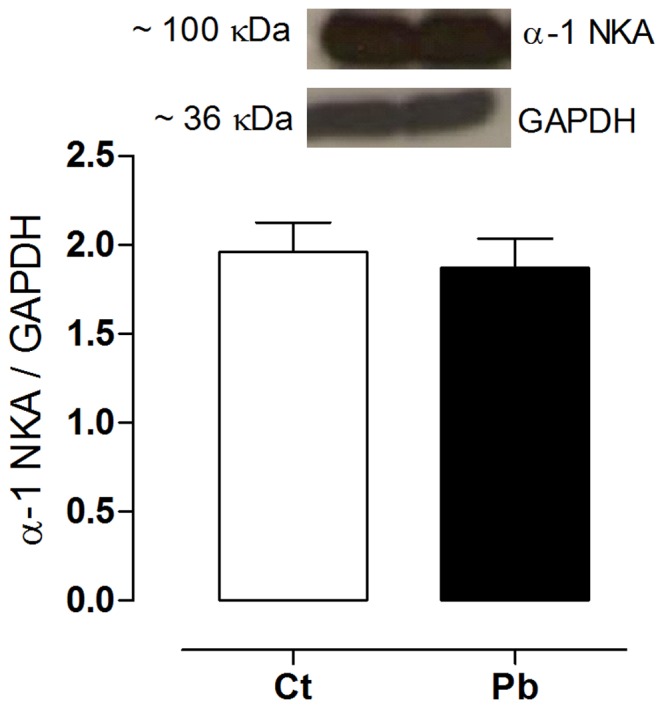
Protein expression levels of the ventricular α-1 Na^+^, K^+^-ATPase subunit. Bands that are representative of the Western blots of the expression of α-1 Na^+^, K^+^-ATPase subunit and GAPDH (Glyceraldehyde 3-phosphate dehydrogenase) are presented at the top of the figure. Results are expressed as the ratio between the area and density of α-1 Na^+^, K^+^-ATPase subunit and GAPDH in the control (Ct) and lead treated (Pb) groups. Number of animals = 7 for each group. *p*>0.05, Ct vs Pb, Student t*-*test.

The sodium-calcium exchanger is an important regulator of calcium cycling in cardiomyocytes, contributing to both contraction and relaxation. Treatment with lead did not alter the expression of this protein, nor did it alter the expression of the cardiac α-1 Na^+^, K^+^-ATPase subunit (Figure 5).

## Discussion

The main results of this study suggested that lead exposure is a potential aggravating factor for the development of hypertension and a risk factor for the development of heart disease. Blood pressure elevations were observed early on in the treatment process and were maintained throughout the course of treatment. In contrast, contractile changes were recorded after 30 days of exposure.

The blood concentrations of lead that were found in the treated group were approximately 13 µg/dL, while the control group had values below the limit of detection of the apparatus. The value in the treated group was lower than the maximum allowable biological index in Brazil (60 µg/dL), according to the “Norma Regulamentadora n° 7 do Ministério do Trabalho” [25]. Therefore, our study demonstrates that short-term lead exposure, which produces blood lead levels that are lower than those that are considered to be safe, is a risk factor for the development of cardiovascular diseases.

Lead exposure promoted the increased deposition of lead in all of the tissues studied compared to the Ct-group, with the exception of the brain (Table 1). Sun et al. (2009) studied the distribution of lead in the blood and organs of mice that had been exposed to lead nitrate. The authors did not investigate the bone deposition of lead, which is the site where the metal has the highest tropism, as indicated by our results. However, they demonstrated that the kidneys were the major site of deposition, followed by the liver, spleen, heart, lungs and brain, which reinforces the results of our study [26].

The systolic arterial pressure became increased during the first week of metal exposure, and remained high over the following weeks (Figure 1). The increase in blood pressure reported here has been well documented in previous studies using exposure models in which animals presented higher blood concentrations of lead or were submitted to longer periods of exposure to this metal [8], [9], [10], [11], [12], [27]. Recently, reports from our laboratory have shown that low blood concentrations of lead (approximately 10 µg/dL) [13] or a single acute exposure (approximately 37 µg/dL) [14] are able to raise the systolic blood pressure of exposed animals. The results of several studies suggest that the pathogenesis of lead toxicity in hypertension is multifactorial. Lead exposure alters calcium homeostasis [28], promotes sympathetic hyperactivity [29], [12], increases the activity of the renin-angiotensin system [10], [14], depresses antioxidant reserves and/or increases the production of reactive oxygen species (ROS), resulting in increased oxidative stress [30], [31], [32], [9], [33]. In addition, lead exposure also alters the vascular response to vasoactive agents [34], [28], [14], promotes endothelial damage, reducing the bioavailability of NO and increasing levels of endothelin [35], [32], [36], [27] and induces kidney damage [37].

Animals treated with lead also showed elevated DAP and HR. These results may be related to increased sympathetic activity, since the literature shows that exposure to lead results in hyperactivity of this system. Boscolo and Carmignani [29] showed that chronic exposure to lead (18 months) induces sympathetic hyperactivity by peripheral and central stimulation increasing the activity of sympathetic vascular receptors (α2) and heart (β1). In addition, it has been reported that the treatment for 10 months with 60 ppm lead acetate promoted increased plasma concentration of adrenaline and noradrenaline [38].

In the present study, the animals had their intraventricular pressure and indices of contractility and relaxation recorded at the end of 30 days of treatment. The assessment of left ventricular (LV) performance revealed that Pb exposure was able to increase LVSP, as well as the left ventricular positive and negative dP/dt. In contrast, treatment with lead did not modify right ventricular parameters (Table 2).

However, haemodynamic measurements are subject to external interference, such as the Anrep effect [39], Frank-Starling mechanism and neurohumoral regulation. To avoid the interference of these systemic influences in myocardial contractility evaluations, we used isolated LV papillary muscles. It is noteworthy that this investigation was unprecedented because there are no reports of studies that investigated the direct effects of chronic exposure to lead on LV myocardial contractility in rats.

Lead exposure did not alter the force or the temporal and inotropic parameters of the LV papillary muscles, indicating that the results obtained *in vivo* and *in vitro* differ. These findings suggest that the cellular mechanisms involved in the genesis of contractile force, such as those involved in the calcium cycle, are regulating positive inotropic stimulation in cardiomyocytes, as visualised in vivo, preventing increases in force in the isolated preparations. Figures 2 and 3 showed protocols that investigated transarcolemmal calcium influx. The results suggested that the cardiomyocytes that were isolated from the animals in the lead-treated group were more permeable to calcium than those isolated from the Ct-group.

The activities of important enzymes that are involved in the regulation of myocardial contractility were measured: cardiac NKA and plasmatic ACE. Lead exposure did not alter the activity of ACE, while the activity of NKA was increased.

To investigate the effects of lead on regulatory mechanisms that govern myocardial contractility, the expression of key proteins that regulate the calcium cycle in cardiomyocytes were quantified. Lead exposure caused a reduction in the expression of SERCA-2 (Figure 4). Reduced expression of SERCA-2 culminates in lower SR calcium uptake and, consequently, reduces the SR calcium content and the availability of this ion for the activation of contractile proteins [40]. This is the reason that the cardiomyocytes that were treated with lead showed no increase in cardiac inotropism, despite showing greater transarcolemmal calcium influx.

These effects of lead on calcium homeostasis in cardiomyocytes were previously unknown. However, studies have reported these increases in red blood cells and correlated lead toxicity in these cells with this factor [41], [42]. The mechanism by which lead acts at the cellular level, favouring calcium influx, remains unclear. However, our results suggest that lead promotes greater calcium influx via L-type calcium channels because the use of verapamil, a blocker of these channels, abolished the increased calcium influx in the muscles of exposed animals (Figure 3).

Lead exposure resulted in increased NKA activity. Lee et al. (2009) showed that activation of NKA results in a cascade of intracellular signalling, activating Src and ERK 1/2, which culminates in the activation of the α-1 subunit of L-type calcium channels [43]. This activation results in increased calcium influx through this channel, which has been proposed as a new mechanism governing calcium signalling in cardiomyocytes. Therefore, because the increased activity of NKA found in our study is consistent with increases in PRC, the concentration-response to calcium (Figure 2) and the responses to tetanic contractions (Figure 3), this may be the mechanism by which lead exposure results in higher calcium influx.

This hypothesis is supported by similar responses that were observed in the post-pause potentiation protocol, as presented in both groups. The PRP manoeuvre was used to indirectly evaluate the release of calcium from the SR [18] and allowed us to conclude that despite the stimulus for calcium-induced calcium release being higher in the Pb-group, the reduced expression of SERCA-2 counterbalanced this response and culminated in similar calcium release and inotropism between the two groups.

Lead exposure reduces the expression of SERCA-2. However, the expression of PBL and its phosphorylated subunits (phospho-Tre^17^-PLB and phospho-Ser^16^-PLB) were not altered (Figure 4). The ratios of the phosphorylated subunits, corrected for total phospholamban (phospho-Tre^17^-PLB/PLB and phospho-Ser^16^-PLB/PLB), were not altered by lead treatment. These ratios were obtained in order to study phospholamban activity and allowed us to conclude that lead exposure did not alter this activity. The result of the combination of reduced SERCA-2 expression and the absence of a compensatory phospholamban response culminated in similar calcium uptake by the SR in both groups, even though the Pb-group was exposed to greater calcium availability.

Reduced SERCA-2 expression may be caused by possible chronic sympathetic stimulation [44]; [45] or may be due to increases in [Ca]i that were promoted by increased calcium influx [46]. Furthermore, it is noteworthy that reduced SERCA-2 expression is considered to be a molecular marker of damage to cardiac performance during the development of left ventricular hypertrophy during heart failure [47]. However, because the participation of the SR did not differ between the two groups, it is likely that the mechanisms that are involved in calcium removal via the sarcolemma are compensating for the increased influx of this ion, thereby avoiding increases in the contractile force.

In conclusion, our study demonstrated that lead exposure resulting in blood lead concentrations that are below the limit recommended by international legislation result in increased blood pressure and heart rate. We also demonstrated for the first time that lead exposure causes changes in calcium handling proteins that may contribute to the deleterious effects of this metal.

### Limitations of the Study

In our laboratory we do not have any equipment for echocardiography and for ionic current measurements. Because of that these protocols were not performed. We evaluate the cardiac mechanical activity by hemodynamic records and isolated papillary muscles contraction and to access information about calcium influx we performed an indirect evaluation using the PRC protocol [20].
